# Novel Deep Convolutional Neural Network-Based Contextual Recognition of Arabic Handwritten Scripts

**DOI:** 10.3390/e23030340

**Published:** 2021-03-13

**Authors:** Rami Ahmed, Mandar Gogate, Ahsen Tahir, Kia Dashtipour, Bassam Al-tamimi, Ahmad Hawalah, Mohammed A. El-Affendi, Amir Hussain

**Affiliations:** 1 College of Computer Sciences and Information Technology, Sudan University of Science and Technology, P.O. Box 407 Khartoum, Sudan; ramiscience@gmail.com; 2 School of Computing, Edinburgh Napier University, Edinburgh EH10 5DT, UK; mandar.gogate@napier.ac.uk (M.G.); a.tahir@napier.ac.uk (A.T.); 3 Department of Electrical Engineering, University of Engineering and Technology, Lahore 54890, Pakistan; 4 Smart Big Data Ltd., London N1 7GU, UK; tami@sbigd.com; 5 College of Computer Science and Engineering, Taibah University, Madina 42353, Saudi Arabia; ahawalah@taibahu.edu.sa; 6 Department of Computer Science, College of Computer and Information Sciences, Prince Sultan University, Riyadh 12435, Saudi Arabia; affendi@psu.edu.sa

**Keywords:** Arabic handwritten, batch normalization, DCNN, dropout, databases

## Abstract

Offline Arabic Handwriting Recognition (OAHR) has recently become instrumental in the areas of pattern recognition and image processing due to its application in several fields, such as office automation and document processing. However, OAHR continues to face several challenges, including high variability of the Arabic script and its intrinsic characteristics such as cursiveness, ligatures, and diacritics, the unlimited variation in human handwriting, and the lack of large public databases. In this paper, we introduce a novel context-aware model based on deep neural networks to address the challenges of recognizing offline handwritten Arabic text, including isolated digits, characters, and words. Specifically, we propose a supervised Convolutional Neural Network (CNN) model that contextually extracts optimal features and employs batch normalization and dropout regularization parameters. This aims to prevent overfitting and further enhance generalization performance when compared to conventional deep learning models. We employ a number of deep stacked-convolutional layers to design the proposed Deep CNN (DCNN) architecture. The model is extensively evaluated and shown to demonstrate excellent classification accuracy when compared to conventional OAHR approaches on a diverse set of six benchmark databases, including MADBase (Digits), CMATERDB (Digits), HACDB (Characters), SUST-ALT (Digits), SUST-ALT (Characters), and SUST-ALT (Names). A further experimental study is conducted on the benchmark Arabic databases by exploiting transfer learning (TL)-based feature extraction which demonstrates the superiority of our proposed model in relation to state-of-the-art VGGNet-19 and MobileNet pre-trained models. Finally, experiments are conducted to assess comparative generalization capabilities of the models using another language database , specifically the benchmark MNIST English isolated Digits database, which further confirm the superiority of our proposed DCNN model.

## 1. Introduction

In the field of handwriting recognition systems (HRSs), digits, characters, and word recognition systems are used in a variety of applications, including bank cheque processing [1,2,3,4,5,6,7,8], office automation [9,10,11,12], document processing [3], document content-based retrieval [13], signature verification [4,7], postal code recognition [1,2,4,5,6] and digital character identification systems. HRS can be carried out both online and offline. Online applications of HRS use digital instruments [2,13], and the identification of characters is dynamically achieved in a sequential manner [13]. The detection also considers key factors, such as pen pressure and velocity [8,14]. Handwriting recognition of scanned documents and digital images is carried out offline [2,14]. Content in these images can be easily converted into editable character codes or words using an HRS, which makes it very useful for text processing applications [8,13,15,16,17,18]. Offline HRS is more challenging and complex when compared to online HRS [8,14]. Benchmark databases [2,5,8,13,14] for both online and offline HRSs, such as SUST-ALT and HACDB [19], are collected from digitized documents compiled by individuals using traditional writing instruments (pen or pencil) [5]. Other databases, such as ADAB and SUST-OLAH (characters and names) [20], are collected from written topics on digital instruments (i.e., smartphones or tablets) [5,21].

**Figure 1:**
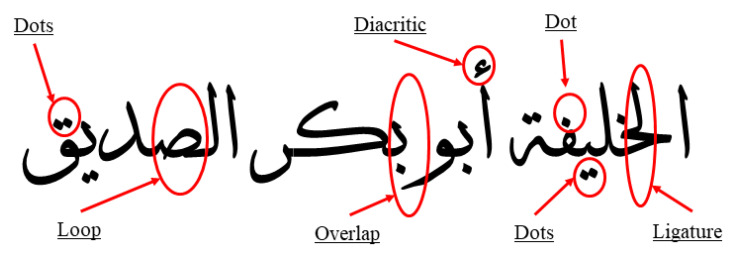
Some of the characteristics of Arabic script writing.

Building a novel robust offline automated HRS for Arabic handwritten scripts remains an open challenge. This is mainly due to several characteristics of the Arabic language, such as its cursive nature, ligatures, overlapping, and diacritical marks [1,3,7,22,23,24,25]. A Few letters in Arabic also have loops, and half of the letters have dots that are used to distinguish between characters. Incorrect recognition of any of these dots can lead to a misrepresentation of the character and, therefore, the whole word [6,26]. To account for variations in the style, size, and shape of characters in human handwriting [11,27,28,29], Figure 1 shows some of the characteristics of writing in Arabic, including cursive writing, ligatures, overlapping, diacritics, dots, and loops.

In contrast to traditional classification/recognition approaches, hierarchical deep neural networks (DNNs) have enabled end-to-end systems for OAHR that do not require pre-processing techniques or manual feature engineering [30]. Multilayer DNN derivatives, such as stacked auto-encoder (SAE), deep belief network (DBN), recurrent neural network (RNN), and convolutional neural networks (CNNs), have proven their high performance and accuracy [10,28,31,32]. The CNN has shown that it outperforms state-of-the-art approaches [33] in various fields, including face recognition, object recognition, and image classification [22,23,34,35,36]. The CNN architecture is a multi-layer feed-forward neural network that adopts the back-propagation algorithm to learn and automatically extract features from high-dimensional and complex data such as images [9,37,38,39,40]. The implementation of a robust CNN model requires the sequence of layers (e.g., convolution, pooling, non-linear transformation, fully connected layers, filters parameters, and loss function formulation) to be defined and, more significantly, requires the use of optimization methods and parameterization [41,42] to improve efficiency. Several techniques were proposed to address overfitting of the DL networks training process, including dropout and batch normalization, and this process has been used to enhance generalization accuracy [43,44,45]. In the case of deep neural network architectures with a number of parameters, overfitting is considered a significant drawback [28]. In order to supervise and control the problem of overfitting, Hinton et al. [46] introduced training with dropout technique where a number of neurons are dropped randomly along with their connections during the training process, and their corresponding weights are not updated [27,28,46,47,48,49,50]; this prohibits units from extreme co-adapting [49], which leads to better generalization capability [22,48,49]. The dropout regularization technique leads to a significant improvement in recognition accuracy on various deep neural network architectures [46,48,51,52,53]. Ioffe and Szegedy [54] proposed batch normalization (BatchNorm) for robust optimization and regularization to enhance model accuracy, accelerate the training of deep networks [22,54,55,56], and at times, eliminate the need for dropout techniques [57] by applying the BatchNorm to the model architecture. They stated that by using this method, several powerful features will be obtained, such as the ability to use significantly higher learning rates, being less cautious with parameter initialization, and achieving the normalization for each training mini-batch [54,57,58].

The CNN model can be exploited in two different ways: either by designing the model manually or automatically and then achieving the training process from scratch [59,60,61,62] or by employing the Transfer Learning (TL) strategies to leverage features from off-the-shelf pre-trained models on bigger databases [30,63]. There are many available state-of-the-arts pre-trained CNN models that have been trained on the ImageNet database [64], such as VGGNet [65], GoogLeNet [66], ResNet [67], InceptionV3 [68], Xception [69], MobileNet [70], and DenseNet [71].The TL technique is used to transfer the acquired knowledge from one or more tasks in the source domain to another task in the target domain [28,63,72,73,74,75] by utilizing a pre-trained network from a source domain that has a considerable amount of training data [74,76], and this helps in boosting the recognition accuracy or reducing training time [74]. The two widely utilized TL strategies are the feature extraction strategy from prior trained data and the fine-tuning strategy of the applied pre-trained network [63,77].

In this paper, we present a robust DCNN sequential model for solving the OAHR problem. The contribution of this work is significant for several reasons. First, our preliminary examinations revealed that a CNN model uses an enormous amount of stacked layers with a high level of generalization to solve the OAHR problem. Second, we conducted experimental studies on six offline Arabic handwritten databases (comprising digits, characters, and words), including the new ALT-SUST databases [78,79]. Third, we used the TL-based feature extraction strategy and carried out experiments on the exploited databases with pre-trained VGGNet-19 and Mobile-Net models for experimental comparison purposes. Fourth, we conducted a comparative study on innovative model performance by pitting the state-of-the-art OAHR approaches against TL-based models evaluated on the aforementioned databases. Finally, we tested the proposed DCNN model’s generalization on other languages, such as the MINST English digits database.

In our study, six different databases were used to recognize digits, characters, and Arabic words. However, most of the current approaches have been used to recognize Arabic characters only.

The remainder of this paper is organized as follows: Section 1 describes the fundamental concepts in the CNN DL training optimization. A review of some of the related work performed in the OAHR area is provided in Section 2. Section 3 shows the general framework of the typical OAHR model. Section 4 presents the proposed DCNN sequential model. The details about the experimental study conducted and the discussions are presented in Section 5. Section 6 summarizes the findings and provides recommendations for future work.

## 2. Related Works

A number of significant approaches have been proposed, and good recognition rates have been reported for specific offline Arabic handwritten databases, especially in the case of digits. However, OAHR is an active research area that always requires accuracy improvement, and accordingly, more generalized and enhanced recognition models are demanded for better accuracy [80,81,82,83,84]. The work presented in this paper was restricted to DL OAHR approaches, and therefore, in this literature review, we focused on reviewing the most recent and competitive DL-related works that solved the OAHR problem.

Elleuch et al. [85] introduced an unsupervised model based on a feature learning approach. The Deep Belief Neural Network (DBNN) approach is composed of two Restricted Boltzmann Machines (RBMs), each with 1000 hidden neurons. The DBNN approach was tested on the HACDB offline Arabic handwritten character database, which contains 6600 shapes (5280 training images and 1320 testing images), and it was normalized to 28 × 28 pixels. The testing on this database obtained 97.9% accuracy using two hidden layers, each with 1000 units. However, the model recognition tests’ generalization scope was not extended to cover the isolated offline Arabic handwritten digits and words; further, the recognition accuracy requires more enhancement.

In another work, Elleuch et al. [37] presented DBN and CNN architectures with a greedy layer-wise unsupervised learning algorithm. Both classifiers were tested and compared on the HACDB databases’ 24 and 66 class labels of character patterns. The HACDB was normalized to 28 × 28 pixels, and its size was expanded ten times using the elastic deformation technique. The DBN achieved an accuracy of 98.33% and 96.36%, and it outperformed CNN, which gained an accuracy of 95% and 85.29% using the 24 and 66 class labels, respectively. However, the generalization scope of the model recognition tests was not extended to cover the isolated offline Arabic handwritten digits and words; further, the recognition accuracy requires more enhancement, especially for the CNN model applied to HACDB (66) database.

A dyadic multi-resolution deep convolutional neural wavelets’ network approach was provided by ElAdel et al. [11] for Arabic handwritten character recognition. The Deep Convolutional Neural Wavelet Network (DCNWN) is based on the Neural Network (NN) architecture, the Fast Wavelet Transform (FWT), and the Adaboost algorithm. The FWT was exploited to extract features of the character based on Multi-Resolution Analysis (MRA) at different abstraction levels.The recognition accuracy of 93.92% was obtained for different IESKarDB database groups, including 6000 segmented characters (2/3 of the database were used in the training phase, and the rest 1/3 in the testing phase). However, the model recognition tests’ generalization scope was not extended to cover the isolated offline Arabic handwritten digits and words; further, the recognition accuracy demands improvement.

Elleuch et al. [86] extended their works by introducing the Deep Belief Neural (DBN) and the Convolutional Deep Belief Network (CDBN) approaches. The authors considered two problems: first, the character recognition (low-level dimensional data) problem for which they used the HACDB database, which contains 6600 shapes (5280 training images and 1320 testing images); second, the word recognition (high-level dimensional data) problem for which they used the IFN/ENIT database of Tunisian towns’ names (26,459 Arabic words). The DBN and the CDBN approaches scored an accuracy of 97.9% and 98.18% on the HACDB database, respectively. The CDBN achieved an accuracy of 83.7% on the IFN/ENIT database. However, the model recognition tests’ generalization scope was not extended to cover the isolated offline Arabic handwritten digits; further, the recognition accuracy requires more enhancement, especially for the CDBN model applied to the IFN/ENITwords database.

A CNN method based on a simple LeNet-5 network was implemented by El-Sawy et al. [9] and evaluated on the MADBase Arabic digit database (60,000 training images and 10,000 testing images). The model achieved an error classification rate (ECR) of 12%. Their work’s drawbacks were the following: they did not try to modify the current LeNet-5 model; the generalization scope of the model recognition tests was not extended to cover the isolated offline Arabic handwritten characters and words; further, the recognition accuracy demands improvement.

Elleuch et al. [28] presented a Support Vector Machine (SVM)-based deep learning model based on Deep Support Vector Machine (DSVM) to classify handwritten Arabic characters. The deep SVM was built using a stack of SVMs, allowing them to automatically extract features from the raw images and recognize them. This model adopted the dropout technique to overcome the overfitting problem. The DSVM model was tested on the HACDB database, which contains 6600 shapes (5280 training images and 1320 testing images), and it achieved a classification accuracy rate of 94.32%. However, the model recognition tests’ generalization scope was not extended to cover the isolated offline Arabic handwritten digits and words; further, the recognition accuracy demands improvement.

Elleuch et al. [27] proposed an offline Arabic handwritten character recognition system using a CNN as a features information extractor and an SVM with Radial Basis Function (RBF) kernel functions as a classifier. The CNN-based SVM was evaluated using the HACDB (with a training set of 5280 images and a test set of 1320 images) and IFN/ENIT databases. The authors utilized the database sets (a) and (b), and they performed pre-processing, including word segmentation into characters, noise reduction, and binarization. In the HACDB database, its training set size was spread out ten times by the elastic deformation technique. The performance was improved when the dropout technique was exploited. The experiments were carried on HACDB with 24 classes, HACDB with 66 classes, and IFN/ENIT with 56 classes, and the results were an ECR of 2.09%, 5.83 %, and 7.05 %, respectively. However, the model recognition tests’ generalization scope was not extended to cover the isolated offline Arabic handwritten digits; further, the recognition accuracy requires more enhancement.

Loey et al. [10] provided a new unsupervised DL method with Stacked Auto-Encoder (SAE) for Arabic digits’ classification. The model’s first and second sparse auto-encoders employed the L2 regularization to enhance the model generalization. The authors tested their model using raw data inputs from the MADBase database (with 60,000 training images and 10,000 testing images), and the testing process achieved an accuracy of 98.5%. However, the model recognition tests’ generalization scope was not extended to cover the isolated offline Arabic handwritten characters and words; further, the recognition accuracy requires more enhancement.

Ashiquzzaman et al. [47] suggested a novel CNN algorithm that uses the Rectified Linear Unit (ReLU) activation function with the dropout technique as a regularization layer to identify numerals in offline handwritten Arabic. The model utilizes many pre-processing operations (e.g., normalized, gray-scaled, inverted to a black background, and a white foreground). The algorithm was tested against the CMATERDB 3.3.1 Arabic handwritten digit database (with 2000 training images and 1000 testing images) and achieved a classification accuracy of 97.4%.However, the model recognition tests’ generalization scope was not extended to cover the isolated offline Arabic handwritten characters and words; further, the recognition accuracy requires more enhancement.

Chen et al. [76] provided a segmentation-free method of RNN with a four-layer bidirectional Gated Recurrent Unit (GRU) network along with a Connectionist Temporal Classification (CTC) output layer and combined it with the dropout technique, which was claimed to improve the system’s generalization ability. The RRN-GRU was designed to identify words in offline handwritten Arabic. The model utilizes many pre-processing operations (e.g., centered, normalized, inverted, re-scaled). The tests were carried out on the IFN/ENIT database with the “abcd-e” scenario, and a classification accuracy rate of 86.49% was obtained. Post-processing was also considered in this language, as it improved the recognition rate. However, the model recognition tests’ generalization scope was not extended to cover the isolated offline Arabic handwritten digits and characters; further, the recognition accuracy demands improvement.

M. Amrouch et al. [87] introduced an integrated architecture based on CNN and the Hidden Markov Model (HMM) classifiers to solve the Arabic handwritten word recognition problem. The CNN-based HMM model was implemented by using a CNN architecture similar to the LeNet-5 as a salient features extractor and the HMM baseline system as a recognizer. That permitted the extraction of relevant characteristics without pre-processing or much emphasis on the feature extraction process. This model was validated using two scenarios of the IFN/ENIT database, named “abc-d” and “abcd-e”, and it gained a recognition accuracy of 88.95% and 89.23%, respectively. However, the model recognition tests’ generalization scope was not extended to cover the isolated offline Arabic handwritten digits and characters; further, the recognition accuracy demands improvement.

Elbashir et al. [19] provided a CNN model for Arabic handwritten character recognition on the SUST-ALT characters database. The pre-processing stage involved normalizing the database to fit in 20 × 20 pixels and then cantering the normalized images into scaled images of 28 × 28 pixels; moreover, these images were inverted to a black background with white foreground colors. The model input data was divided into 70% for training and the reset into 30% for both testing and validation data. The obtained recognition accuracy was 93.5%. However, the model recognition tests’ generalization scope was not extended to cover the isolated offline Arabic handwritten digits and words; further, the recognition accuracy demands improvement.

A framework of Convolutional Deep Belief Network (CDBN) was proposed by Elleuch et al. [88] for recognizing low/high-level dimensional data. The overfitting problem was reduced using the data augmentation and dropout regularization techniques to improve model performance. The authors validated the CDBN architecture on two Arabic handwritten script databases, the HACDB and IFN/ENIT. They achieved an accuracy rate of 98.86% on the HACDB characters database (with a training set of 5280 samples and a test set of 1320 samples). For the IFN/ENIT words database, they obtained an accuracy rate of 91.55% using protocol 1 (a, b, and d for training and e for testing) and a recognition accuracy of 92.9% using protocol 2 (a, b, and c for training and d for testing). However, in terms of generalization capability, each database problem was solved by different structures. Moreover, the model recognition tests’ scope was not extended to cover the isolated Arabic handwritten digits; further, the recognition accuracy demands improvement.

In work presented by Ashiquzzaman et al. [89], the authors proposed an amendment to their same approach presented in [28]. This amendment included introducing data augmentation (rotation, noise, zooming randomly, shifting horizontally and vertically, and points outside the boundaries being filled according to the nearest point) to prevent overfitting, thereby changing the activation from ReLU to the Exponential Linear Unit (ELU) in order to prevent the vanishing gradient problem. The exploited database was the CMATERDB 3.3.1 digits data type, and it was divided at a ratio of 2:1 for training and testing purposes. The database images were pre-processed by inverting their colors. After implementing all these changes, the proposed model obtained an accuracy of 99.4%. However, the model recognition tests’ generalization scope was not extended to cover the isolated offline Arabic handwritten characters and words.

Mustafa et al. [90] presented a CNN model for recognizing SUST Arabic names (words) holistically. The authors employed the dropout and batch normalization techniques from the posted model’s structure, which helped their model be more suitable for solving the high-level dimensional problem. They pre-processed the database’s raw images by eliminating the surrounding white spaces, applying a downscaling of 28 × 56 pixels, and converting to a black background and white foreground. The achieved a recognition accuracy of 99.14%. However, in terms of generalization capability, the proposed CNN model was evaluated only on 20 classes out of 40 classes, which is considered a significant drawback; further, experiments were not conducted to test the scope of the model recognition tests with respect to other databases’ data types, such as isolated Arabic handwritten characters and digits.

In terms of pre-processing and accuracy, the findings and limitations of the existing OAHR methods are summarized in Table 1.

## 3. OAHR General Framework

**Figure 2:**
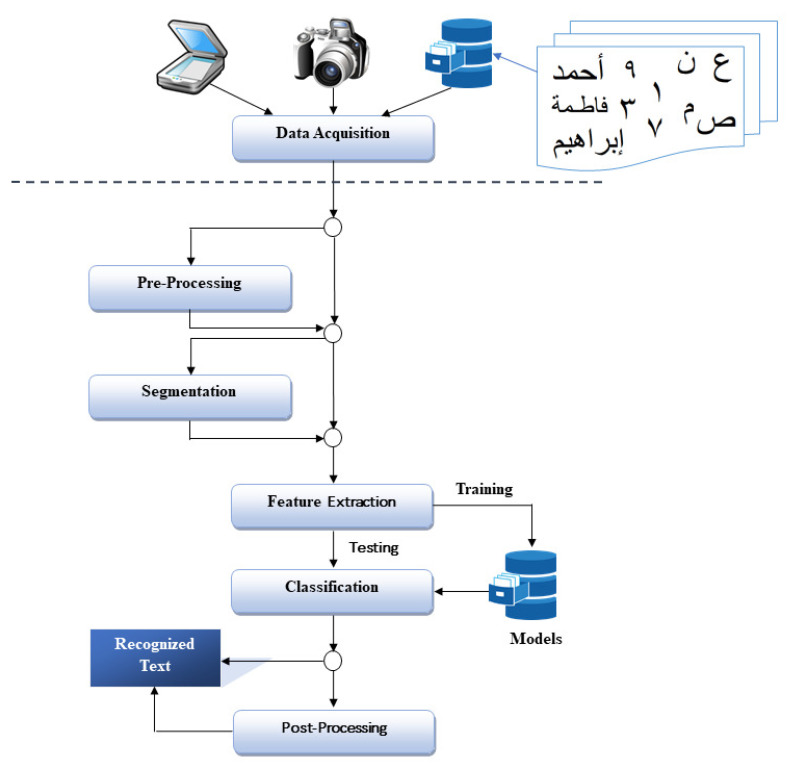
Offline Arabic handwritten recognition system general framework.

Figure 2 illustrates the general framework of OAHR, which involves six phases, namely data acquisition, pre-processing, segmentation, feature extraction, classification, and post-processing. The researchers can utilize all, subset, or merged stages in their systems [3,25]. Each phase is described as below in brief:

### 3.1. Data Acquisition

Image acquisition is the first phase of the Arabic handwriting recognition system. Cameras and scanners are generally adopted for capturing or acquiring offline text images. Using the optical scanner device is the most appropriate way of obtaining text images because it provides the ability of implementing automatic adjustment, binarization, and enhancements such as low noise on the images, which helps to improve the system’s recognition accuracy [7]. For research purposes, mostly the ready and publicly published databases are used.

### 3.2. Pre-Processing

The quality of the input text image can influence the accuracy of the recognition process [91]. The role of the pre-processing phase is to enhance the quality of raw text image by clearing irrelevant information and, then, to provide a clean text image that can be proper and efficient for the next phases [3,14,92,93]. The pre-processing task can be achieved by applying many techniques that can deal with skew/slant detection and correction, thinning, binarization, noise removal, thresholding, normalization [3,92,93], resizing and compression, etc. [14,94]. One or more pre-processing techniques can be used based on the degree of the text image quality [1,95] and according to the targeted OAHR design.

### 3.3. Segmentation

The segmentation phase constitutes a sensitive and critical task because any fault, such as misplaced segmentation, over-segmentation, or under segmentation, impacts the entire recognition process [3,91]. The role of this stage is to split a text image into pixel segments, a piece of Arabic words (PAWs), or line segments [93], and this comprises text lines, words, sub-words, characters, and sub-characters. The segmentation operation has three strategies, including the classical systems where character-like properties are used to identify the segments [93], the segmentation-based systems where the word is segmented into characters and primitives or segmented after the recognition [3], and the holistic or segmentation free systems where the recognition process is done on the entire word without splitting [1,3,91,93]. Numerous segmentation methods are not sufficient for offline Arabic handwriting tasks due to its cursive nature, the presence of dots, diacritics, overlaps, and ligatures, which cause more difficulty during character segmentation, and accordingly, additional researches are required in this area [3].

### 3.4. Feature Extraction

Feature extraction is the process of extracting the most relevant and non-redundant attributes from the text image raw data [1,3,14,92] and then converting them into a vector of features [9,91,95]. The different types of feature methods are classified as structural features, statistical features, and feature space transformations [8,94,95]. Structural features describe the geometrical and topological characteristics of a text image pattern, such as directions, strokes, endpoints, the intersection of line segments, number of diacritical marks, loops, types of dots, zigzag, arcs, concavities, etc. Statistical features are extracted from the pixels’ statistical distribution, and they describe the set of characteristic measurements of a text image pattern, such as number of loops, segments, branching and crossing points, and diacritics and their positions [95], and they have low complexity and high speed [1,3,8,94]. Feature space transformations convert the pixel representation to a more compact form with less feature vector dimensionality [7,8,94] among the methods involving principal component analysis (PCA), Walsh Hadamard Transform, Fourier Transform, Wavelets, Hough Transform, Gabor Transform, Rapid Transform, and Karhunen Loeve Expression and moments [1,3,8,9,94,95].

### 3.5. Classification

The classification phase is the decision-making process of the recognition system, and its role relies on recognizing and allocating an input feature with a class label or membership scores for digits, characters, or words to the correct related defined classes [1,3,92], so that the texts in images are transformed into a computer understandable form [92], and the performance of a classifier depends on the quality of the extracted features. In the literature, the categories of numerous recognition approaches, including template matching [92] and the structural, statistical, stochastic, and hybrid approaches, are obtained by combining multiple classifiers [95]. For Arabic handwritten recognition, many classifiers have been used, such as k-nearest neighbors (kNN), HMM, SVM, and ANN [1,3,93,94,95,96]. In recognition systems, this phase is achieved by selecting a suitable recognition method and then employing two processes: the training process, which uses extracted features to train the classifier to build the appropriate models, and the testing process, which uses the previously generated models.

### 3.6. Post-Processing

After the completion of the classification phase, the final post-processing phase can be optionally added to enhance the proposed system’s accuracy and reliability by refining the decisions taken by the previous stage and then minimizing the classifier recognition error rate. This task can be achieved by using the Arabic linguistic knowledge level, which could be on many levels, such as character, lexical, morphological, syntactic, higher semantic, and discourse levels [93]. Many methods have been introduced in this context, such as the implementations of the Damerau-Levenshtein distance methods for solving the string correction problem and the n-gram methods used for statistical language models [95].

In light of the OAHR’s general framework demonstrated six phases and since the targeted databases are already collected, our proposed DCNN model will utilize only three phases, including pre-processing, feature extraction, and classification.

## 4. Proposed DCNN Model

**Figure 3:**
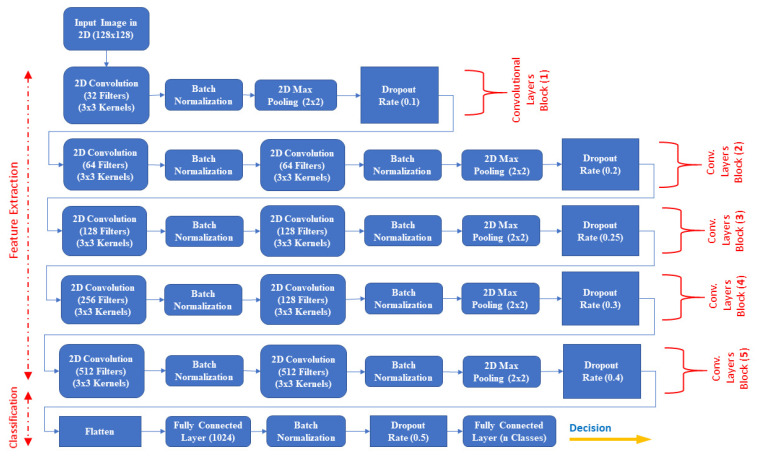
The proposed DCNN architecture for oahr.

In this section, we illustrate the implementation of the proposed modern deep ConvNets architecture that is built from blocks of alternating convolution layers, normalization layers, max-pooling layers, and dropout, followed by some fully connected layers. CNN is a deeply hierarchical, multi-layer neural network with trainable weights and biases [47] trained with the back-propagation algorithm [27,37,47]; it was inspired by the information processing in the human brain [9]. The CNN algorithm was constructed from an automatic feature extractor and a trainable classifier that was used to efficiently learn complex, high-dimensional data [27,28] and high-level features from a labeled training data [23]; it was used to build hierarchical representations from raw data [28] and then for solving image classification problems [23] such as OAHR. In this study, the proposed DCNN sequential model was used primarily with 34 building blocks, including one input layer, one output layer, five stacked convolutional layers blocks, and two fully connected hidden layers for nonlinear classification. Figure 3 shows the innovative DCNN architecture that shows the building blocks of two main phases of the OAHR system: the feature extraction phase and the classification phase.

### 4.1. Design Methodology

Determining a suitable design for deep machine learning network architecture (e.g., CNN) is a bit of a “black art” because each given problem (database) requires certain adjustments; accordingly, the performance of the learning process is critically sensitive to the chosen architecture design [61]. Therefore, the chosen CNN model’s performance relies on setting the design structure, the data representation, and the training process controlled through several hyper-parameters [60]. The structural hyper-parameters encompass decisions that should carefully be tuned, such as depth of the network (e.g., number of convolutional and fully-connected layers), number of filters, layer type, number of units per layer and in fully-connected layers, stride size, pooling locations, sizes, dropout rates, batch normalization, and learning rates [60,61]. The hyper-parameters’ number and types increase in the deeper and complex modern models [60]. On the other hand, deciding the appropriate combination of hyper-parameters for a specific recognition task is challenging due to the unclarity of their interaction and their impact on model performance [61]. There are two methodical ways of designing CNN network architecture: the hand-crafted design and the automated design [59,60,61,62]. The CNN hand-crafted architecture’s design requires considerable human expertise and effort in manually tuning the CNN architecture’s hyper-parameters [61,62] in the problem domain [62]. Thus, a set of trial and error combinations must be achieved to ensure efficient design due to the massive number of architectural design choices, which are considered challenging and time-consuming processes [59,61]. Unlike modern CNN hand-crafted models such as Mobilenet, Squeezenet, and Shufflenet, the hand-crafted CNN-based VGGnet, GoogLeNet, and ResNet models are consuming a vast amount of resources and time in the training process [59]. On the other hand, the automated design methods for finding an optimal arrangement of the network building blocks that achieve the best performance becomes a solution for solving the problem of needing human experts and avoiding the expert trial-and-error procedure [59,60]. The automated methods, such as grid search, random search, or formulating the selection of appropriate hyper-parameters as an optimization problem, are employed for finding the best combination of CNN architecture’s hyper-parameters that yields a better design [61]. Hence, we headed toward designing the DCNN model architecture from scratch using the hand-crafted methodology for this proposed work. We started by building a simple CNN model with one block, and then we built up the network complexity and got deeper, as shown in Figure 3, by trying varying combinations of hyper-parameters and seeking better performance improvement. The target contribution of providing well-generalized (recognizing isolated Arabic digits, characters, and words) and well-performed (high and competitive recognition accuracy and precision) added more challenge, efforts, and time to the hyper-parameters’ manual tuning process, which led us to commit to using five blocks of convolutionals. The results of the experiments on stacked DCNN blocks are revealed in the “results and discussion” section.

### 4.2. Feature Extraction Phase

For all the convolutional layers in this model, the original input dimensions will remain intact by adopting a padding option called “same”. The model description is provided in the following way: block one of the convolutional features extraction started with the first convolutional layer that possesses 32 feature maps, each with a trainable kernel size of (3 × 3) pixels and a ReLU activation function of the neurons. It can extract features from the input raw image whether it is Grayscale, RGB, or RGBA of size 128 × 128 or 331 × 94, depending on the database types used in the experiments. The second layer is the batch normalization layer, which was added after the convolutional layers; it used the mean and variance to limit their output away from the region of saturation [22]. The maximum sub-sampling or pooling layer with a pool size of 3 × 3 was used as the third layer for reducing the dimensionality of the feature maps. The regularization or dropout layer was used in the next layer to reduce overfitting, and it is configured to randomly and temporarily remove 10% of the neurons. Relatively, the configuration of the following repeated convolution layers was represented by the next four stacked blocks in order.

### 4.3. Classification Phase

After all of these convolutionals, we started the classification layers of the model with a flattened layer to transform the resulting two-dimensional matrix of features into a single vector; subsequently, it was fed into the first fully connected layer that selected 1024 neurons and the ReLU activation function. Next, the batch normalization layer was added; a dropout of 0.5 followed it. Finally, a second full connection layer was used as the highest level of the architecture. It was configured to the actual number of neurons corresponding to the targeted Arabic database class labels. For this final layer, the Softmax activation function was used to return a list of probability-like predictions between (0 and 1) for each of the class labels.

## 5. Experiments and Discussion

We conducted tests on the recognition of offline Arabic handwritten digits, characters, and words by employing standard stacked Deep ConvNets along with the batch normalization technique and dropout technique for improved efficiency. We examined the proposed DCNN architecture on multiple databases, including the MADBase, CMATERDB, HACDB, SUST-ALT (digits), SUST-ALT (characters), and SUST-ALT (names). The results are presented and discussed in the next subsection.

### 5.1. Details About Arabic Handwritten Databases

This section presents two categories of the databases employed in these experiments; these are the commonly used benchmark databases (MADBase, CMATERDB, and HACDB) and the newly used SUST-ALT databases for Arabic handwritten digits, characters, and names. The MADBase digits benchmark database is a modified version of the ADBase, which is composed of 70,000 greyscale digits images (60,000 training images and 10,000 test images) written by 700 writers. It has the same format as the Latin database (MNIST) [9,10]. The CMATERDB version 3.3.1 is another Arabic handwritten digits database benchmark that contains 3000 unique samples of 32 × 32 pixel RGB bitmap images [47]. The HACDB version 2.0 characters benchmark database contains 6600 greyscale character images, which have generated two forms for 66 shapes (58 shapes of characters and eight shapes of overlapping characters) [27,28,37,85,86,97]. The newly used SUST ALT (Sudan University of Science and Technology – Arabic Language Technology group) database includes several Arabic handwritten databases. The digits database and the isolated letters database (34 classes) are created from scratch, while the source of the names database (40 classes) is the SUST graduation certificate application forms [19,33]. Table 2, Table 3 and Table 4 present some raw samples of the experiments on databases based on their types while covering digits, characters, and words from the Arabic handwritten databases, respectively.

### 5.2. Experimental Setup and Pre-Processing

The computer for the experimental setup had the following: a Windows 10 operating system and Intel(R) Core(TM) i7-7700HQ CPU @ 2.80 GHz, 16 GB RAM, Nvidia Geforce (GTX 1050 Ti) with a 4 GB RAM Graphics card that supported the parallel computing platform – Compute Unified Device Architecture (CUDA) version 7.1.4 with Graphics Processing Unit (GPU) enabled. The experimental model was implemented with Google’s TensorFlow, an open-sourced framework that uses Keras’s higher-level API framework built on top of the TensorFlow for machine learning models’ implementation and deployment as well as the Python open-sourced DL library for programming. The model compilation is an efficiency step that is required after defining the model. We specified the parameters tailored to train the established networks, which were selected as follows: for optimizing network training, the experiments proved that the RMSprop algorithm had performed better in this model; for evaluating the network, the (Categorical_Crossentropy) multi-class logarithmic loss reduction function was used along with the metric function (accuracy) that was used to judge the performance of the model. The model training, for all the experiments, was accomplished by assigning training function parameters with the following values: epochs number was 200 and batch size was 128. The (ReduceLROnPlateau) function was used to reduce the learning rate during the training process with the following customized argument values: monitor was val_acc, patience was 1, verbose was 1, factor was 0.5, and learning rate was 0.00001. The model was constantly fed with a database that needed to be split into parts, including validation, testing, and training; the latter one was configured to be 80% of the overall size of the training database. In regard to the pre-processing of data, better recognition was achieved by applying the augmentation and resizing processes automatically to the input database images without any handcraft. It was observed that the model interface loads the raw images as it is for the SUST-ALT names database with 331 × 94 pixel size, unlike the digits (MADBase, CMATERDB, SUST-ALT numbers) and characters (HACDB, SUST-ALT letters) databases that are resized to 128 × 128 pixels. The augmentation of the image data process makes the model more robust by creating more data from the existing ones by applying only a slight transformation using the shift/range property for each image with width and height values of 0.15.

### 5.3. Results and Discussion

The following sub-sections discuss the employed classification evaluation criteria, performance of the proposed hand-crafted DCNN model on the targeted Arabic databases, its generalization on a non-Arabic database (i.e., English handwritten digits), and finally, a comparative study of its performance with the pre-trained VGGNet-19 and MobileNet models as well as the state-of-the-art DL approaches in the field.

#### 5.3.1. Evaluation Criteria

The quantitative evaluation of the supervised classification learning approach’s performance linked to its prediction capability of unseen and independent data is fundamental in estimating the quality of learning approaches and learning models in machine learning [98]. Obtaining an optimal generative classifier is subject to selecting a suitable evaluation metric during the classification training for measuring its quality [98]. Thus, there may be many typical evaluation metrics for measuring new classifiers’ performances, accuracy, and classification error [98,99], which have been noticed through related works in the previous section. However, the accuracy and error rate are considered to be less distinctive, less informative, strongly biased to favor the majority class data (i.e., the imbalance problem), Refs. [98,99] and sensitive to class skews; accordingly, in such cases, other metrics such as recall, precision, and F-measure are more appropriate as benchmark measurements [98]. Therefore, different evaluation metrics can evaluate different classifiers’ characteristics for specific classification algorithms [99]. Five evaluation metrics based on the confusion matrix for multi-class prediction were exploited for measuring the proposed model, and the pre-trained model performances were accuracy, error rate, recall, precision, and F-measure. Accuracy measured the ratio between the correct predictions and the total number of predictions made [99]. Misclassification error or error classification rate (ECR) measured the ratio between incorrect predictions and the total number of predictions [99]. Recall or sensitivity: measured the ratio between correctly the classified positive patterns and the total positive patterns [99,100]. Precision measured the ratio between the correctly predicted positive patterns and the total predicted positive patterns [99,100]. F-Measure or F-score represented the harmonic mean of recall and precision [99]. The following formulas (1)–(5) showed the definitions of ECR, accuracy, recall, precision, and F-measure, respectively:

(1) $$ECR=\frac{FP\;+\;FN}{TP\;+\;TN\;+\;FP\;+\;FN}$$

(2) $$Accuracy=\frac{TP\;+\;TN}{TP\;+\;TN\;+\;FP\;+\;FN}$$

(3) $$Recall=\frac{TP}{TP\;+\;FN}$$

(4) $$Precision=\frac{TP}{TP\;+\;\;FP}$$

(5) $$F-measure=2\;\times \frac{\;Precision\;\times \;Recall}{Precision\;+\;\;Recall}$$

where: *TP* = True Positive or correct positive prediction; *TN* = True Negative or correct negative prediction; *FP* = False Positive or incorrect positive prediction; *FN* = False Negative or incorrect negative prediction.

#### 5.3.2. Incremental Approach to Proposed Model Design

In this work, we sought to improve the OAHR model’s capabilities and performance in the field of DL by providing a more generalized model that can recognize many types of offline Arabic handwriting databases, including isolated numbers, letters, and words. The following experiments’ details showed the block-wise evolvement of the design of the proposed hand-crafted DCNN model. The three performance measurements, i.e., accuracy, precision, and training time, considered the main factors of assessing the process of stacking architecture blocks for each database individually and all databases as a whole. Table 5 shows that the accuracy and precision metrics of the MADBase digits database were improved for each stacked block, and their values were relatively close. Thus, the model’s architecture with five stacked blocks performed better than other architectures with fewer stacked blocks in terms of accuracy, precision, and training time. It should be noted that the three performance metrics of precision, recall, and F-measure for each experiment for this database as well as the followed databases’ experiments revealed identical values, and this could be attributed to the equality of the obtained FN and FP numbers.

Similarly, Table 6 shows that the performance metrics of accuracy and precision of the CMATERDB digits database were ameliorated for each stacked block, but their values were divergent. Accuracy value can be high but precision value low as in the case stated here, which meant that the model performed well, but the obtained results were spread [13]. Our target was to achieve both high accuracy and high precision. Again, during this experimental phase, the model’s architecture with five stacked blocks performed better than the other ones with fewer stacked blocks in terms of accuracy, precision, and training time.

The accuracy and precision metrics in Table 7 were incrementally improved for each stacked block, and their values were relatively close for those architectures with three, four, and five blocks. The model architecture of four blocks had better accuracy and precision than the model with five blocks, but on the other hand, it was too slow. The model with five blocks was faster than all other models for the SUST-ALT digits database.

Table 8 shows that the models with four and five blocks had the best accuracy and precision metrics on the HACDB characters database, but their values were divergent, and the precision was low. Although the four-block model had better performance metrics, it was slower than the five-block model.

The accuracy and precision metrics of the conducted experiments on the SUST-ALT digits database were divergent; on the other hand, they were incrementally boosted as shown in Table 9, and their scored values were relatively close to those architectures with three, four, and five blocks. In this case, the four-block model had better accuracy and precision than the five-block model, but on the other hand, it was too slow. Again, the five-block model was faster than all the other models with fewer stacked blocks.

Finally, the experiments on the SUST-ALT words database showed systematic growth of the precision and accuracy through all architectures as illustrated in Table 10, and in this case, the five-block model scored the best performance metric values. Although the precision was high, it was still considered relatively low when compared to the obtained accuracy. In terms of training time, it was slower than the three- and four-block models. It is worth mentioning that the used environment’s GPU was resistant to the training batch size in this category of experiments due to the big size of the actual input word’s image size. The models’ architectures with one, two, three, four, and five blocks exploited batch sizes of 16, 32, 64, 96, and 128, respectively, which indicated that the five-block model keeps the batch size unchanged, which ensures the unification of the final DCNN model’s hyper-parameters remains intact.

Since the four-block and five-block models showed outstanding performances in comparison with the models with fewer blocks and as these obtained performance measurement values were relatively close, we summarized the assessment factors, as shown in Table 11, to determine the best model for OAHR problems. From this summary, it is clear that there was a relative balance between the four-block model and the five-block model in terms of best accuracy and best precision, and the conclusive measurement factor between them was the training time in which the five-block model had the least training time. Besides that, it was observed that the five-block model can support working with large samples’ sizes without affecting the model’s hyper-parameters (i.e., training batch size). For these reasons, we approved using the DCNN model with five blocks.

From the automatic statistical readings, mainly from the generated experiments’ confusion-matrix, it was evident that our powerful novel model achieveed high accuracy with reasonable training time; Table 12 shows the details of the final DCNN model’s experiments, which were categorized based on the database type.

For the digits category experiments, the CMATERDB, SUST-ALT digits, and MADBase scored a high accuracy rate of 98.85%, 99.65%, and 99.88%, respectively, for training, a validation accuracy rate of 98.96%, 99.34% and 99.73%, resectively, and a testing/prediction accuracy rate of 99.72%, 99.82%, and 99.91% in the given order. The recognition of the MADBase outperformed the other databases in this category in terms of training, validation, and testing accuracy rates. For the characters’ category experiments, the HACDB database gained a training accuracy rate of 97.87%, a validation accuracy rate of 97.65%, and a high testing/prediction accuracy rate of 99.91%. The SUST-ALT characters database achieved a training accuracy rate of 98.73%, a validation accuracy rate of 97.97%, and a testing/prediction accuracy rate of 99.86%. HACDBhas also outperformed the other databases in this category in terms of training, validation, and testing accuracy rates. For the category of the words’ high-level dimensional data, the model proved that it could work significantly better compared to other low-level dimensional databases (digits and characters) along with the words’ holistic recognition; in this respect, it scored a high accuracy rate of 99.48% for the training, a validation accuracy rate of 99.13%, and a testing accuracy rate of 99.95%. The summary of the proposed model’s quantitative recognition performance metrics and other databases on this benchmark, including precision, recall, F-measure, and accuracy, are shown in Table 13.

#### 5.3.3. Comparative Study

In this sub-section, we achieved two types of comparative study: experimental and analytical. The comparison factors that were employed were training time, precision, and accuracy.

(A) Experimental Comparative Study

Two new sequential models were designed to achieve the desired TL experiments with top classification layers, which were the same as the proposed DCNN model’s classification layers (see Figure 3). The very deep VGG-19 pre-trained model was attached to the first sequential model without its top layers, and all of its trainable layers were frozen to make it act as a features extractor during the training process. The same design was applied to the second model using the light-weighted MobileNet pre-trained model. The experiments’ environment was typical to the environment setup, databases’ pre-processing, and architecture’s hyper-parameters as illustrated in Section 5.2 except for the SUST-ALT (words) database. In the VGGNet-19 model experiment using SUST-ALT (words), the batch size was downgraded to 64 due to the GPU limitations with respect to the pre-trained model’s structure. On the other hand, in the MobileNet model experiment, resizing the SUST-ALT (words) database’s samples to square dimensions was required by the model, and in this case, we used the preferred image size of 128 × 128. The training processes were carried out using the TL-based feature extraction training strategy where the source task was either the VGGNet-19 or MobileNet pre-trained model and the target tasks were the six databases (one database at a time for each experiment). Table 14 illustrates the obtained results of the VGGNet-19 pre-trained model on all the six Arabic databases that were used. This very deep pre-trained model showed considerable accuracy, but it was relatively below the accuracy achieved by the proposed DCNN model. In terms of the preciseness, the precision metric scored low values compared to the proposed model, especially for the HACDB, SUST-ALT (characters), and SUST-ALT (words) databases, which emphasized the spreading of the results. In contrast to the proposed model, the VGGNet-19 consumed enormous training time. As a result, the DCNN model was more accurate, precise, and faster than the VGGNet-19 model.

Table 15 shows the results of the MobileNet pre-trained model’s experiments on all the six Arabic databases used. Compared to the proposed DCNN model, this light-weighted pre-trained model scored very low recognition accuracy and displayed the worst preciseness. The precision metric achieved minimal values for the consumed databases expected for the MADBase and SUST-ALT (words) databases, which were relatively reasonable; thus, these results were considered widespread. In contrast to the proposed model, the MobileNet consumed less training time; however, the MADBase database equalled the DCNN model’s training time. As a result, the proposed DCNN model was much accurate and precise than the MobileNet model, and on the other hand, it was slower.

(B) Analytical Comparative Study

Table 16 illustrates the proposed system’s comparative analytical study against other state-of-the-art DL approaches by utilizing the experiments’ offline handwritten Arabic benchmark database. Our obtained results were sufficiently more significant for all exploited databases than the other DL methods that use the CNN technique or other different classification techniques. In this comparative study, we used only the accuracy metric due to the absence of the other classification performance metrics (e.g., recall, precision, and f-measure) and the training time required by the authors’ approaches.

#### 5.3.4. Generalization Tests on Offline English Handwritten Digits

Moreover, in the context of measuring the applicability of our model’s generalization in recognizing other languages’ isolated scripts, we conducted the tests on the MNIST English digits database (with 60,000 training images and 10,000 testing images) using the proposed DCNN model. The experiment required 295 minutes for the training process, and the outstanding accuracy of 99.94 % that was obtained revealed the model’s remarkable potential. On the other hand, the obtained quantitative recognition performance metrics’ precision, recall, and F-measure on this benchmark had the same value of 0.9968%. These results surpassed that of many recent dedicated state-of-the-arts methods [101,102,103,104] evaluated on this database.

## 6. Conclusions and Future Work

This paper demonstrated the effectiveness of exploiting a powerful DCNN system to address challenging OAHR tasks applied to different types of benchmark databases. The proposed model was combined with several standard stacked deep ConvNets along with pooling, dropout, batch normalization, and fully connected layers. The dropout and batch normalization and regularization layers were shown to improve the performance of the model and significantly minimize error rate. We demonstrated that the proposed approach can effectively deal with high-dimensional data by automatically and contextually extracting the best features. Comparative experimental outcomes were seen to be promising with a reported testing accuracy of 99.91%, 99.72%, 99.91%, 99.82%, 99.86%, and 99.95% obtained for MADBase, CMATERDB, HACDB, and three types of SUST-ALT databases, including digits, characters, and words, respectively.

Aside from the comparative analytical study, a further experimental study was conducted on the benchmark Arabic databases, by exploiting transfer learning (TL)-based feature extraction which demonstrated the superiority of our proposed model in relation to state-of-the-art VGGNet-19 and MobileNet pre-trained models. The model’s generalization was also proven on the MNIST English isolated digits database with an excellent accuracy of 99.94% and a precision of 99.68%.

Future research will attempt to further improve the generalization capability of the proposed model, especially for real databases that have an insufficient number of training samples, such as HACDB. This could include employing deeper models with more than five building blocks, novel semi-supervised learning (e.g., [105]), dependency rules-based deep learning (e.g., [106]) and transfer/multi-task learning techniques (e.g., [107]. Other state-of-the-art deep and reinforcement learning models (e.g., [108] can also be exploited and comparatively evaluated on a range of real world databases.

## Figures and Tables

**Table 1:** Summary of reviewed related works.

Literature/ Year	Findings	Outline
Elleuch et al. [85]/2015	The DBNN method obtained an ECR of 2.1% and an accuracy of 97.9% on the HACDB characters database.	Generalization was not tested for Arabic digits and words. Accuracy requires enhancement.
Elleuch et al. [37]/2015	The DBN method obtained an ECR of 1.67% and 3.64% and an accuracy of 98.33% and 96.36% on the HACDB database with 24 characters and the HACDB database with 66 characters, respectively.	Generalization was not tested for Arabic digits and words. Accuracy requires enhancement.
Elleuch et al. [37]/2015	The CNN method obtained an ECR of 5% and 14.71% and an accuracy of 95% and 85.29% on the HACDB database with 24 characters and the HACDB database with 66 characters, respectively.	Generalization was not tested for Arabic digits and words. Accuracy requires enhancement.
ElAdel et al. [11]/2015	The DCNWN method obtained an ECR of 2.1% and an accuracy of 93.92% on the IESKarDB characters database.	Generalization was not tested for Arabic digits and words. Accuracy requires enhancement.
Elleuch et al. [86]/2015	The DBN method obtained an ECR of 6.08% and an accuracy of 97.9% on the HACDB database with 66 characters.	Generalization was not tested for Arabic digits and words. Accuracy requires enhancement.
Elleuch et al. [86]/2015	The CDBN method obtained an ECR of 1.82% and 16.3% and an accuracy of 98.18% and 83.7% on the HACDB (24) characters and the IFN/ENIT words databases, respectively.	Generalization was not tested for Arabic digits and characters. Accuracy requires enhancement.
El-Sawy et al. [9]/2016	The CNN method obtained an ECR of 12% and an accuracy of 88% on the MADBase digits database.	Generalization wa not tested for Arabic characters and words. Accuracy requires enhancement.
Elleuch et al. [28]/2016	The DSVM method obtained an ECR of 5.68% and an accuracy of 94.32% on the HACDB (66) characters database.	Generalization was not tested for Arabic digits and words. Accuracy requires enhancement.
Elleuch et al. [27]/2016	The CNN-SVM method obtained an ECR of 2.09%, 5.83%, and 7.05% and an accuracy of 97.91%, 94.17%, and 92.95% on the HACDB database with 24 characters, the HACDB database with 66 characters, the IFN/ENIT (56) words databases, respectively.	Generalization was not tested for Arabic digits. Accuracy requires enhancement.
Loey et al. [10]/2017	The SAE method obtained an ECR of 2.6% and an accuracy of 98.5% on the CMATERDB 3.3.1 digits database.	Generalization was not tested for Arabic characters and words. Accuracy requires enhancement.
Ashiquzzaman et al. [47]/2017	The CNN method obtained an ECR of 1.5% and an accuracy of 97.4% on the MADBase digits database.	Generalization was not tested for Arabic characters and words. Accuracy requires enhancement.
Chen et al. [76]/2017	The RRN-GRU method obtained an ECR of 13.51% and an accuracy of 86.49% on the IFN/ENIT words database.	Generalization was not tested for Arabic digits and characters. Accuracy requires enhancement.
M. Amrouch et al. [87]/2018	The CNN-based HMM method obtained an ECR of 11.05% and 10.77% and an accuracy of 88.95% and 89.23% on the IFN/ENIT words database with “abd-e” protocol and the IFN/ENIT words database with “abcd-e”, respectively.	Generalization was not tested for Arabic digits and characters. Accuracy requires enhancement.
Elbashir et al. [19]/2018	The CNN method obtained an ECR of 6.5% and an accuracy of 93.5% on the SUST-ALT characters database.	Generalization was not tested for Arabic digits and words. Accuracy requires enhancement.
Elleuch et al. [88]/2019	The CDBN method obtained an ECR of 1.14%, 8.45%, and 7.1% and an accuracy of 98.86%, 91.55%, and 92.9% on HACDB database with 66 characters, the IFN/ENIT words database with “abd-e” protocol, and the IFN/ENIT words database with “abc-d” protocol, respectively.	Generalization was not tested for Arabic digits. Accuracy requires enhancement.
Ashiquzzaman et al. [89]/2019	The CNN method obtained an ECR of 0.6% and an accuracy of 99.4% on the CMATERDB 3.3.1digits database.	Generalization was not tested for Arabic characters and words.
Mustafa et al. [90]/2020	The CNN method obtained an ECR of 0.86% and an accuracy of 99.14% on the SUST-ALT words database.	Generalization was not tested for Arabic digits and characters.

**Table 2:** Raw samples of digits from Arabic handwritten databases.

Number Name	Machine Form	MADBase Database	CMATERDB Database	SUST-ALT Digits
Zero	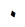	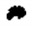	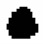	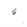
One	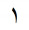	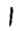	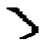	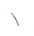
Two	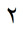	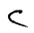	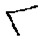	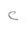
Three	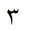	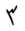	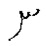	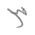
Four	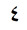	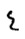	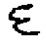	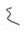
Five	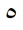	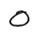	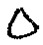	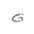
Six	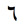	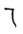	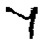	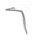
Seven	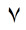	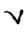	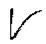	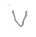
Eight	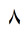	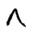	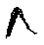	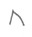
Nine	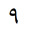	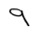	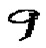	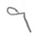

**Table 3:** Raw samples of characters from Arabic handwritten databases.

Character Name	Machine Form	HACDB Database	SUST-ALT Characters Database
Alif	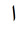	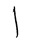	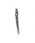
Raa	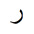	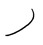	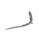
Seen	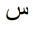	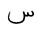	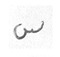
Saad	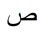	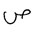	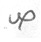
Ayn	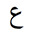	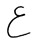	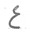
Faa	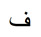	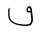	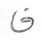
Meem	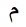	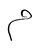	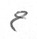
Noon	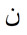	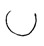	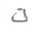
Haa	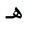	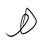	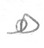
Waw	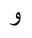	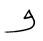	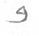

**Table 4:** Raw samples of words from Arabic handwritten databases.

Name in English	Machine Form	SUST-ALT Names Database
Ahmed	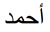	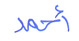
Ali	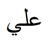	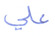
Ebraheem	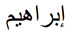	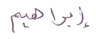
Taha	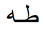	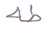
Soliman	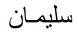	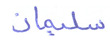
Eman	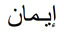	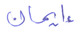
Fatema	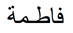	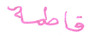
Rian	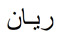	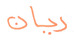
Marwa	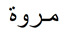	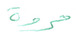
Samah	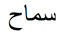	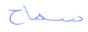

**Table 5:** The block-wise performance metrics of the proposed DCNN model on the MADBase digits database.

Stacked Blocks Count	Training Time (in Minutes)	Precision	Recall	F-Measure	Accuracy
One	586	0.991	0.991	0.991	0.9982
Two	628	0.994	0.994	0.994	0.9988
Three	654	0.9945	0.9945	0.9945	0.9989
Four	717	0.9951	0.9951	0.9951	0.99902
Five (Final Model)	548	0.9953	0.9953	0.9953	0.99906

**Table 6:** The block-wise performance metrics of the proposed DCNN model on the CMATERDB digits database.

Stacked Blocks Count	Training Time (in Minutes)	Precision	Recall	F-Measure	Accuracy
One	24	0.96833	0.96833	0.96833	0.99367
Two	25	0.975	0.975	0.975	0.99
Three	26	0.97	0.97	0.97	0.994
Four	27	0.98542	0.98542	0.98542	0.99708
Five (Final Model)	22	0.98608	0.98608	0.98608	0.99722

**Table 7:** The block-wise performance metrics of the proposed DCNN model on the SUST-ALT digits database.

Stacked Blocks Count	Training Time (in Minutes)	Precision	Recall	F-Measure	Accuracy
One	288	0.96061	0.96061	0.96061	0.99212
Two	350	0.9885	0.9885	0.9885	0.9977
Three	491	0.99283	0.99283	0.99283	0.99857
Four	343	0.99391	0.99391	0.99391	0.99878
Five (Final Model)	282	0.99107	0.99107	0.99107	0.99821

**Table 8:** The block-wise performance metrics of the proposed DCNN model on the HACDB characters database.

Stacked Blocks Count	Training Time (in Minutes)	Precision	Recall	F-Measure	Accuracy
One	52	0.70833	0.70833	0.70833	0.99116
Two	53	0.93561	0.93561	0.93561	0.99805
Three	59	0.95909	0.95909	0.95909	0.99876
Four	60	0.97197	0.97197	0.97197	0.99915
Five (Final Model)	53	0.96967	0.96967	0.96967	0.99908

**Table 9:** The block-wise performance metrics of the proposed DCNN model on the SUST-ALT characters database.

Stacked Blocks Count	Training Time (in Minutes)	Precision	Recall	F-Measure	Accuracy
One	393	0.82687	0.82687	0.82687	0.98982
Two	573	0.95338	0.95338	0.95338	0.99726
Three	400	0.9733	0.9733	0.9733	0.99843
Four	611	0.97799	0.97799	0.97799	0.99871
Five (Final Model)	344	0.97591	0.97591	0.97591	0.99858

**Table 10:** The block-wise performance metrics of the proposed DCNN Model on the SUST-ALT Words (Names) Database.

Stacked Blocks Count	Training Time (in Minutes)	Precision	Recall	F-Measure	Accuracy
One	1079	0.67788	0.67788	0.67788	0.98389
Two	1345	0.96213	0.96213	0.96213	0.99811
Three	504	0.98725	0.98725	0.98725	0.99936
Four	508	0.9895	0.9895	0.9895	0.99948
Five (Final Model)	534	0.99038	0.99038	0.99038	0.99952

**Table 11:** Summary of the performance measurement factors of the four-block model and the five-block model.

Database/Type	Four Blocks			Five Blocks		
	Best Accuracy	Best Precision	Less Training Time	Best Accuracy	Best Precision	Less Training Time
MADBase (Digits)	No	No	No	Yes	Yes	Yes
CMATERDB (Digits)	No	No	No	Yes	Yes	Yes
SUST-ALT (Digits)	Yes	Yes	No	No	No	Yes
HACDB (Characters)	Yes	Yes	No	No	No	Yes
SUST-ALT (Characters)	Yes	Yes	No	No	No	Yes
SUST-ALT (Words)	No	No	Yes	Yes	Yes	No

**Table 12:** Results of the proposed DCNN model’s experiments on different offline handwritten Arabic databases.

Database Name/Type	Training Time (Minutes)	Training Loss (%)	Training Accuracy (%)	Validation Loss (%)	Validation Accuracy (%)	ECR (%)	Accuracy (%)
MADBase/Digits	548	0.46	99.88	1.41	99.73	0.09	99.91
CMATERDB/ Digits	22	3.43	98.85	6.66	98.96	0.28	99.72
SUST-ALT/ Digits	282	1.15	99.65	3.24	99.34	0.18	99.82
HACDB/ Characters	53	6.84	97.49	9.25	96.97	0.09	99.91
SUST-ALT/ Characters	344	3.71	98.73	9.18	97.97	0.14	99.86
SUST-ALT/ Words	534	1.86	99.48	3.97	99.13	0.05	99.95

**Table 13:** Proposed DCNN model’s performances metrics with respect to different offline Arabic handwritten databases.

DatabaseName	DatabaseType	Precision	Recall	F-Measure	Accuracy
MADBase	Digits	0.9953	0.9953	0.9953	0.99906
CMATERDB	Digits	0.98608	0.98608	0.98608	0.99722
SUST-ALT	Digits	0.99107	0.99107	0.99107	0.99821
HACDB	Characters	0.96967	0.96967	0.96967	0.99908
SUST-ALT	Characters	0.97591	0.97591	0.97591	0.99858
SUST-ALT	Words	0.99038	0.99038	0.99038	0.99952

**Table 14:** VGGNet-19 model’s performance metrics of different offline Arabic handwritten databases.

Database Name	Database Type	Training Time (in Minutes)	Precision	Recall	F-Measure	Accuracy
MADBase	Digits	964	0.9921	0.9921	0.9921	0.99842
CMATERDB	Digits	32	0.97667	0.97667	0.97667	0.99533
SUST-ALT	Digits	423	0.98755	0.98755	0.98755	0.99751
HACDB	Characters	70	0.91439	0.91439	0.91439	0.99741
SUST-ALT	Characters	606	0.93002	0.93002	0.93002	0.99588
SUST-ALT	Words	892	0.866	0.866	0.866	0.9933

**Table 15:** MobileNet model’s performance metrics with respect to different offline Arabic handwritten databases.

Database Name	Database Type	Training Time (in Minutes)	Precision	Recall	F-Measure	Accuracy
MADBase	Digits	548	0.8221	0.8221	0.8221	0.96442
CMATERDB	Digits	19	0.41167	0.41167	0.41167	0.88233
SUST-ALT	Digits	263	0.29088	0.29088	0.29088	0.85818
HACDB	Characters	42	0.19697	0.19697	0.19697	0.97567
SUST-ALT	Characters	330	0.1456	0.1456	0.1456	0.94974
SUST-ALT	Words	275	0.915	0.915	0.915	0.95458

**Table 16:** Performance comparisons with other state-of-the-art approaches.

Literature	Method Name	Database Name (Classes)	Database Type	ECR/ WER	Accuracy
Elleuch et al. [85]	DBNN	HACDB (66)	Characters	2.10%	97.90%
Elleuch et al. [86]	DBN	HACDB (66)	Characters	2.10%	97.90%
Elleuchet al. [86]	CDBN	HACDB (66)	Characters	1.82%	98.18%
Elleuch et al. [37]	CNN	HACDB (66)	Characters	14.71	85.29%
Elleuch et al. [37]	DBN	HACDB (66)	Characters	3.64%	96.36%
Elleuch et al. [27]	CNN based-SVM	HACDB (66)	Characters	5.83%	94.17%
Elleuch et al. [28]	DSVM	HACDB (66)	Characters	5.68%	94.32%
Elleuch et al. [88]	CDBN	HACDB (66)	Characters	1.14%	98.86%
**Present Work**	**DCNN**	**HACDB (66)**	**Characters**	**0.09%**	**99.91%**
El-Sawy et al. [9]	CNN	MADBase (10)	Digits	12%	88%
Loey et al. [10]	SAE	MADBase (10)	Digits	1.50%	98.50%
**Present Work**	**DCNN**	**MADBase (10)**	**Digits**	**0.09%**	**99.91%**
Ashiquzzaman et al. [47]	CNN	CMATERDB (10)	Digits	2.60%	97.40%
Ashiquzzaman et al. [89]	CNN	CMATERDB (10)	Digits	0.60%	99.40%
**Present Work**	**DCNN**	**CMATERDB (10)**	**Digits**	**0.28%**	**99.72%**
Elbashir et al. [19]	CNN	SUST-ALT (40)	Words	6.50%	93.50%
Mustafa et al. [90]	CNN	SUST-ALT (20)	Words	0.86%	99.14%
**Present Work**	**DCNN**	**SUST-ALT (40)**	**Words**	**0.05%**	**99.95%**

## Data Availability

Not applicable.
